# Development of a 3D printed device to support long term intestinal culture as an alternative to hyperoxic chamber methods

**DOI:** 10.1186/s41205-017-0018-z

**Published:** 2017-09-20

**Authors:** Matheus O. Costa, Roman Nosach, John C.S. Harding

**Affiliations:** 1 Department of Farm Animal Health, Faculty of Veterinary Medicine, Utrecht University, Yalelaan 7, 3584 CL Utrecht, The Netherlands; 2 Department of Large Animal Clinical Sciences, Western College of Veterinary Medicine, University of Saskatchewan, 52 Campus drive, Saskatoon, SK S7N 5B4 Canada

**Keywords:** 3D Printing, IVOC, In vitro organ culture, Intestinal explant, Colon explant, Pig colon culture, Tissue culture device

## Abstract

**Background:**

Most interactions between pathogenic microorganisms and their target host occur on mucosal surfaces of internal organs such as the intestine. In vitro organ culture (IVOC) provides an unique tool for studying host-pathogen interactions in a controlled environment. However, this technique requires a complex laboratory setup and specialized apparatus. In addition, issues arise when anaerobic pathogens are exposed to the hyperoxic environment required for intestinal culture. The objective of this study was to develop an accessible 3D–printed device that would allow manipulation of the gas mixture used to supply the tissue culture media separately from the gas mixture exposed to the mucosal side of explants.

**Results:**

Porcine colon explants from 2 pigs were prepared (*n* = 20) and cultured for 0h, 8h, 18h and 24h using the device. After the culture period, explants were fixed in formalin and H&E stained sections were evaluated for histological defects of the mucosa. At 8h, 66% of samples displayed no histological abnormalities, whereas samples collected at 18h and 24h displayed progressively increasing rates of superficial epithelial erosion and epithelial metaplasia.

**Conclusio﻿ns:**

The 3D–design reported here allows investigators to setup intestinal culture explants while manipulating the gas media explants are exposed to, to support tissue viability for a minimal of 8h. The amount of media necessary and tissue contamination are potential issues associated with this apparatus.

**Electronic supplementary material:**

The online version of this article (10.1186/s41205-017-0018-z) contains supplementary material, which is available to authorized users.

## Background

The intestinal mucosa is an active host-microorganism interaction interface, and when innate and humoral immune defenses mechanisms fail, infection and disease result. Understanding virulence mechanisms of pathogenic microorganisms, and their consequences, provides an alternative target for disease prevention and mitigation [1]. In vitro models provide advantages for studying virulence mechanisms, given the controlled environment and ease of sample collection. In vitro organ culture (IVOC) involves excision of a small volume of tissue from a healthy donor, and its subsequent culture in appropriate media in the laboratory for a number of days. IVOCs preserve important tissue characteristics, such as different cell types and tissue architecture, while providing a controlled and accessible environment to study host-pathogen interactions [2, 3]. To prevent acidification of the culture media and maintain tissue viability, intestinal IVOCs require a hyperoxic gas environment [4–6]. Sealed chambers are usually placed within incubators for culture, an expensive setup that requires extensive bench space and is potentially an explosion hazard due to the high oxygen content [6, 7]. In addition, this setup becomes a challenge when studying host interactions with anaerobic pathogens, due to the hyperoxic environment. Here, we describe and evaluate a 3D–printed device to allow the use of different gas mixtures in the culture media, compared to the mucosal aspect of the tissue.

## Methods

### 3D–Device design, printing and assembling

**Fig. 1:**
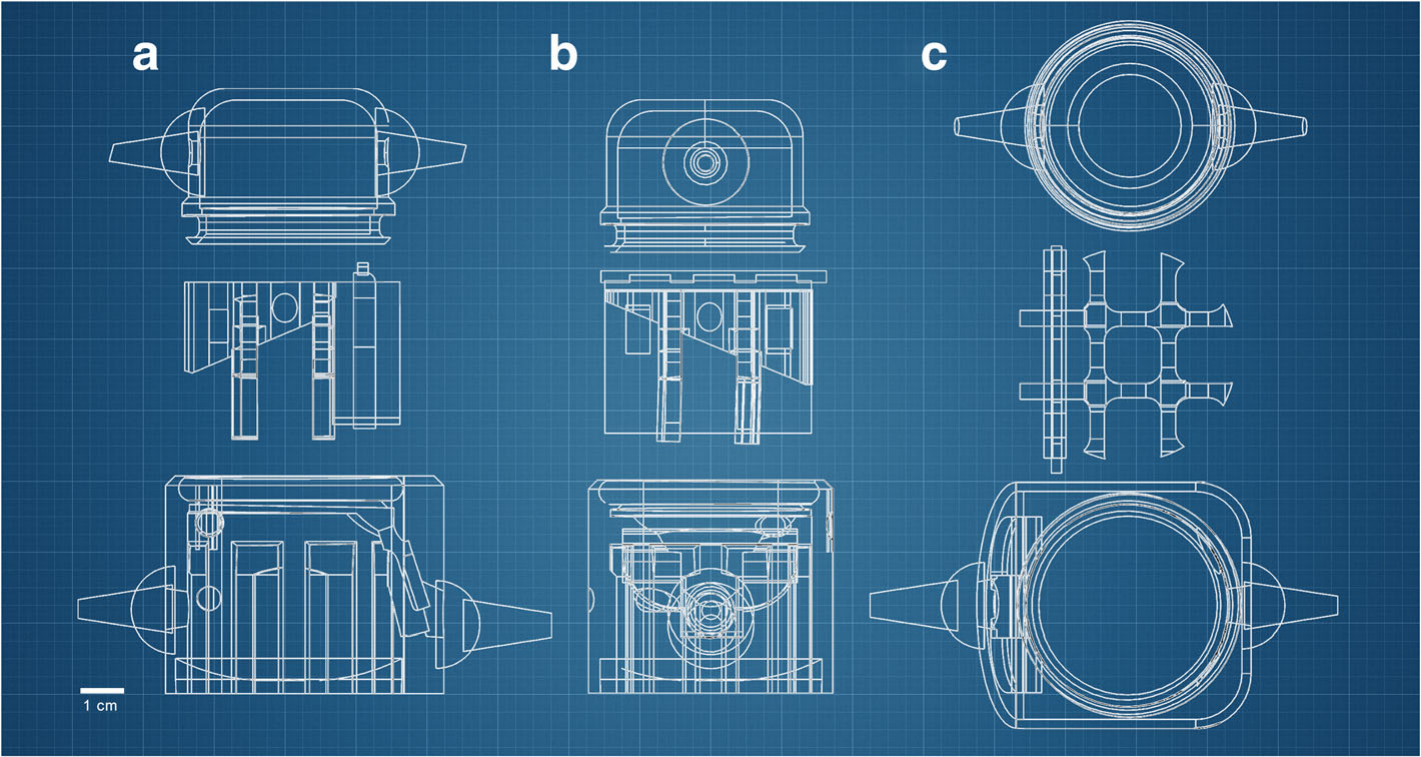
Blueprint of the culture device depicting views from side (**a**), front (**b**), top (**c**)

**Fig. 2:**
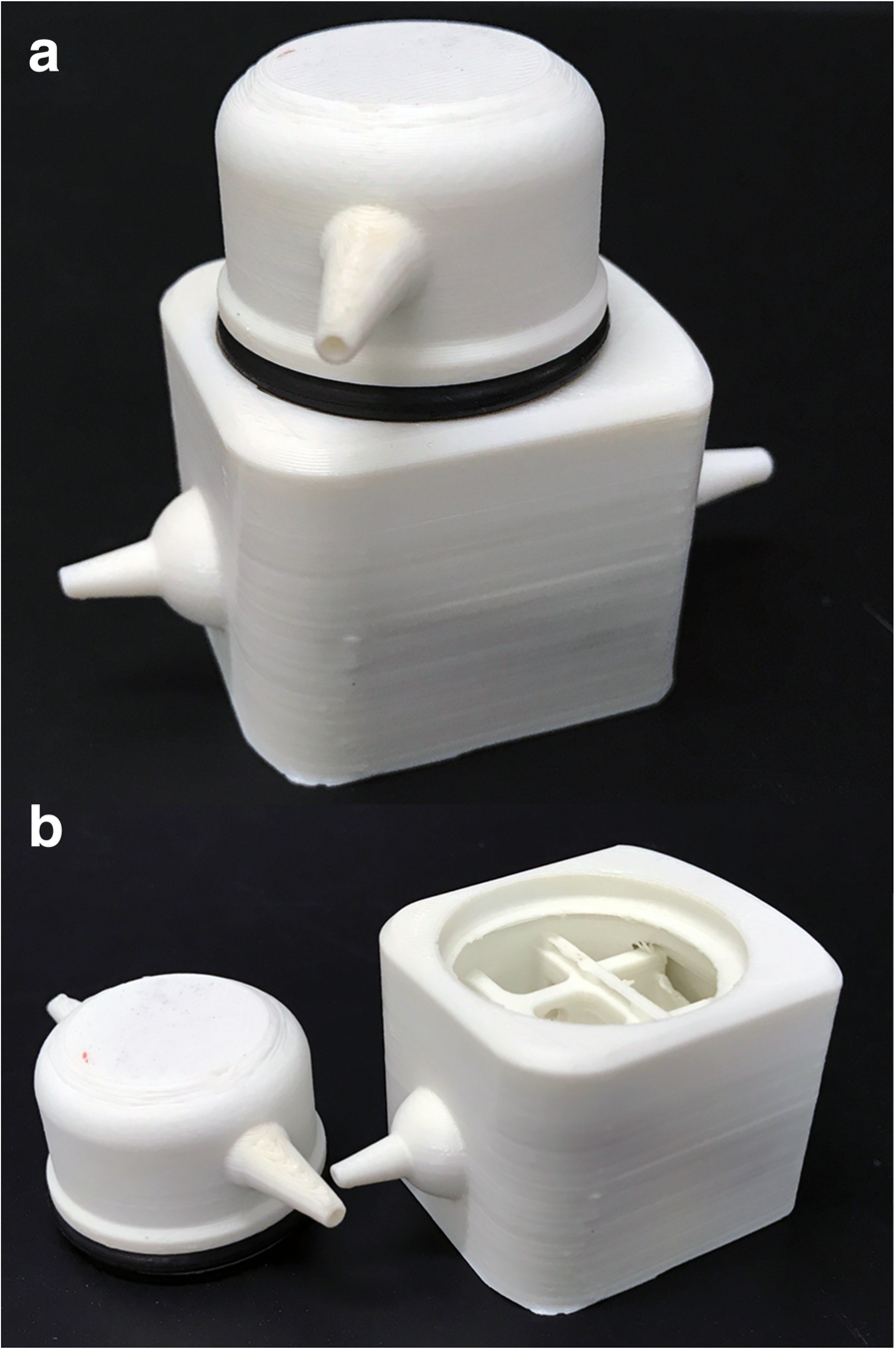
3D–printed culture device. The top is reversibly attached to the bottom using a latex O-ring. The device is shown assembled ready for use (**a**) and disassembled (**b**) showing the tissue support grid where explants are placed for culture

A 3D modelling software (123D Design, AutoDesk Inc., CA) was used to design and develop the 3D–printed device in silico. The device is divided into two parts; one for tissue support and liquid media flow (bottom), and one for the gas media flow (top). To reversibly seal the top-bottom junction, a rubber O-ring of 14.5 mm diameter was used (Fig. 1). The design was exported as two STL files and printed at a commercial printing facility in Saskatoon, Saskatchewan (Fig. 1, Additional file 1). Printing was performed on a MakerBot Replicator Mini + printer (MakerBot Industries, NY), at 500 μ resolution, using an acrylonitrile butadiene styrene (ABS, MG Chemicals, BC) filament. Post-printing cleaning consisted of manually removing any non-used filaments and other printing debris. Due to the small size of the device, 3D–printing the four hollow tubing adapters was impractical. Thus, a 0.3969 mm (1/64″) drill bit was used to create an opening in each adapter, from the tip to the inside of the device. The rubber O-ring (4.4 cm diameter) was placed on the top part of the device to seal the top-bottom junction (Fig. 2).

### Colon segment collection and explant preparation

**Fig. 3:**
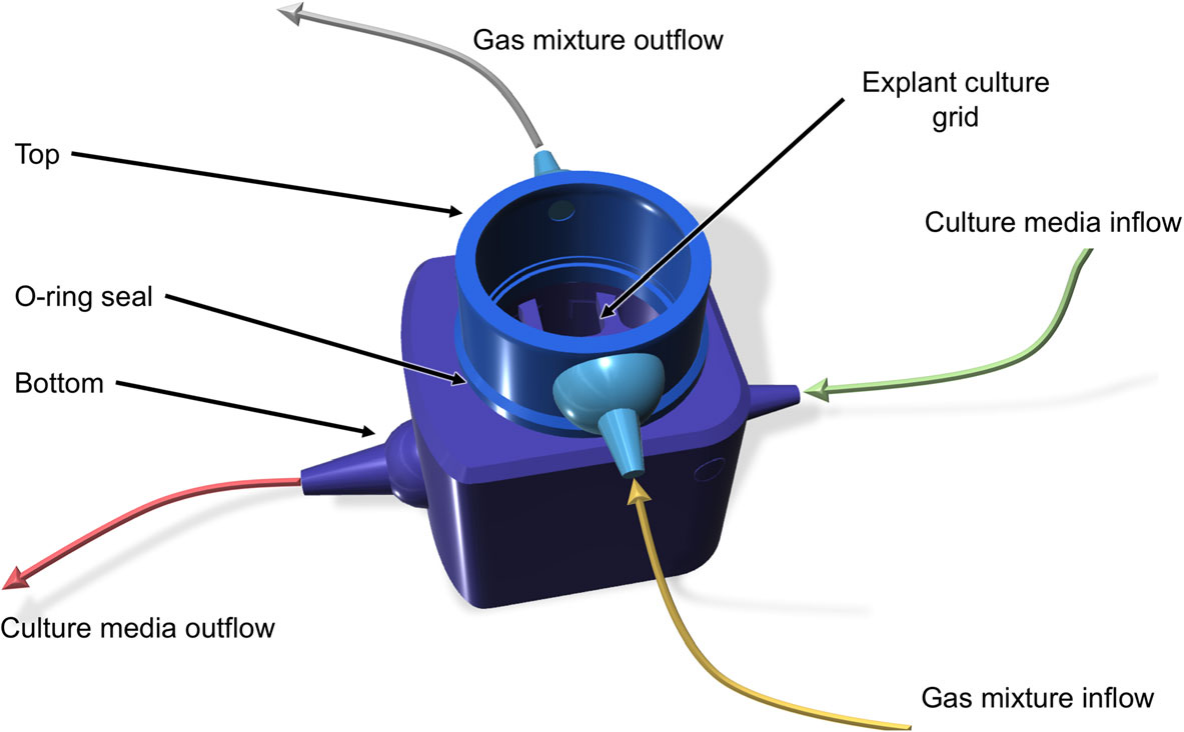
Culture device suggested setup. The top of the cap was intentionally removed to allow visualization of the culture grid. Suggested placement of liquid and gas media tubing is shown by coloured arrows

**Fig. 4:**
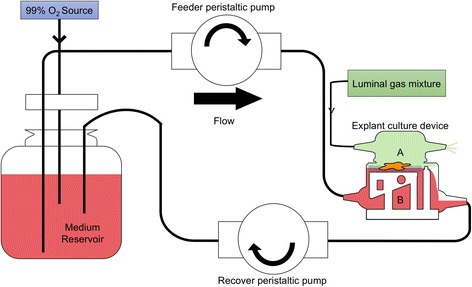
Diagram of culture setup. Tissue culture media (red) is placed in a reservoir bubbled with 99% O_2_ gas. A feeding peristaltic pump is used to pull media out of the reservoir into the culture device bottom (B), bathing the explant (orange form). A vertical wall in the bottom portion of the chamber maintains the depth of the liquid media at the tissue support grid A recovery pump is used to pull the media out of the device back into the media reservoir. A gas mixture of choice (green) flows with low pressure into the top part of the device (A) from a compressed source

Pigs (*n* = 2) were humanely euthanized at approximately 8 weeks of age for reasons unrelated to this study. Tissue culture procedures and culture media preparation have been previously described [5]. Once explants (0.5 cm × 0.5 cm, *n* = 3/device) were ready for culture, they were placed mucosa side up on top of a polystyrene foam (VWR, Mississauga, ON) on the designated grid within the 3D–printed device (Fig. 3. The foam was sterilized by fully submersing it in a 70% ethanol / 30% MilliQ water solution 24 h prior to explant culture, followed by 1 h drying period on a sterile surface within a Biosafety cabinet. Prior to tissue placement, the 3D–device was connected to two electric peristaltic pump (Fig. 4, Cole-Parmer, AO-73160-32, Chicago, IL) and flushed with a commercial disinfecting solution (Virkon, DuPont, Wilmington, DE) for 5 min. Next, sterile NaCl solution (0.85%) was used to wash the 3D device and attached tubing for another 5 min. All tubing used was standard sterile intravenous tubing (Lifeshield – primary set, Hospira, Lake Forest, IL). Finally, feeding pump was set at 10 mL/min, connected to the bottom part of the 3D–device (Fig. 4) and a reservoir containing tissue culture media (100 mL/device) supplemented with antibiotics (KBM-Gold Ca2+ and Phenol red free BulletKit (catalog number 195769), Lonza, Walkersville, MD). The second pump, the recover pump, was set at a higher rate to prevent the device from over flowing (15 mL/min). The liquid media was bubbled with medical grade mixture of 99% O_2_, 1% CO_2_ flowing from a compressed gas cylinder connected to a regulator, at a 3 L/min rate. For this experiment, the top gas inlets received atmospheric air. Both culture media reservoir and 3D–device were kept immersed in 2 cm high distilled water bath at 37 °C.

### Explant fixation and analysis of H&E stained sections

To assess tissue viability, explants were placed in 10% buffered formalin for tissue fixation at 0 h (*n* = 2), 8 h (*n* = 6), 18 h (n = 6) and 24 h (n = 6). Within 48 h, the explants were paraffin embedded and stained using Haematoxylin and Eosin (H&E), as per routine protocols. Histological analysis of explant morphology was carried out on H&E stained sections by an observer (MC) blinded to the identity of the samples using optical microscopy at 20× magnification. The presence and quality of superficial epithelium, crypts, goblet and necrotic/apoptotic cells within crypts were recorded.

## Results and discussion

This study describes a low-cost, 3D–printed device used for intestinal explant culture without the need of a hyperoxic chamber or incubator. 3D–printing required approximately 2 h including manual removal of leftover filament and printing debris. No leakage was observed after 24 h of continuous liquid media circulation through the circuit, and the media was maintained constantly at 37 °C.

**Fig. 5:**
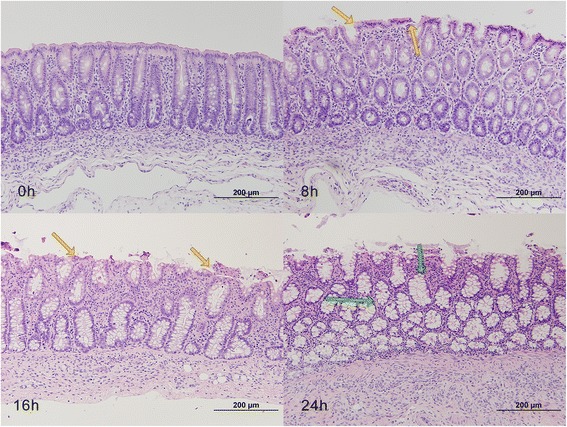
H&E stained sections of porcine colon explants cultured using the 3D–printed device. Overall, normal tissue architecture is observed until 24 h after initial culture setup, with progressively increasing sloughing off the superficial epithelial (yellow arrow) layer beginning at 18 h. Distension of crypt glands is observed at 24 h (green arrow)

Porcine colon explants sampled at 0 h, immediately before placement on the culture device, did not show any histological abnormalities. A total of 66% (4/6) of samples collected at 8 h displayed normal architecture, with the presence of crypts, goblet cells and normal epithelial turnover. Sloughing off the superficial epithelial layer increased at 18 h (50%, 3/6) of samples) and 24 h (66%, 4/6), possibly as a response to the culture environment. At both latter time points, a catarrhal exudate accumulated on the apical side of the explants. After 24 h in culture, explants also displayed distension of glands, perhaps due to the lack of clearance of accumulated mucus from the apical surface. Representative H&E stained explant sections are shown on Fig. 5.

Although explants were only kept viable for a maximum of 24 h in this experiment, this period is suitable for the majority of studies focused at pathogen-host interactions [8–10]. In addition, the ability to manipulate the gas media that explants are exposed can benefit studies aimed at intestinal physiology [11] and nutrition [12] by simulating more closely the luminal gas environment normally observed in vivo. Although providing continual circulation, this setup uses considerably more culture media compared to a 6-well culture plate setup (3 mL vs 100 mL) to continuously provide explants with nutrients and with metabolite clearance by dilution. If culture takes place outside a biosafety cabinet, the setup is prone to contamination.

## Conclusions

The design described here consistently supported porcine colon explants for up to 8 h with minimal disruption of tissue viability. It also provides an alternative culture method that does not require the use of a hyperoxic chamber, and is particularly suited for situations where a gas environment other than atmospheric is needed on the mucosal aspect of the explant.

## Additional files

Additional file 1: 3D printed device design (STL files). (STL 2224 kb)

Additional file 2: ﻿3D printed device design 2 (STL files). ﻿(STL 3978 kb)

## Abbreviations

H&E: Haematoxilin and eosin.
IVOC: In vitro organ culture
